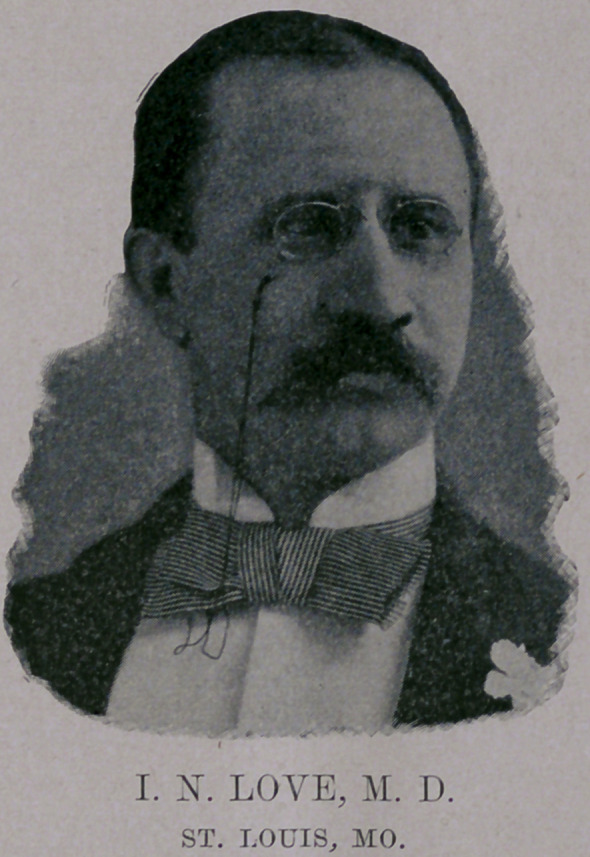# Articles

**Published:** 1900-07

**Authors:** 


### TEXAS MEDICAL JOURNAL.

AUSTIN, TEXAS.

A MONTHLY JOURNAL OF MEDICINE AND SURGERY.

EDITED AND PUBLISHED BY

F. E. DANIEL, M. D., AND S. E. HUDSON, M. D.

Published Monthly at Austin, Texas, by Drs. Daniel and Hudson. Subscription price $1.00 a year in advance.

Eastern Representative: John Guy Monihan, St. Paul Building, 220 Broadway, New York City.

Official organ of the West Texas Medical Association, the Houston District Medical Association, the Austin District Medical Society, the Brazos Valley Medical Association, the Galveston County Medical Society, and several others.

I. N. LOVE, M. D.

ST. LOUIS, MO.

THIS is a shadow of the corporeal Dr. Love; no art can paint,—nor pen portray the intellectual,—the spiritual Love. But his genial, generous heart,—too generous for his own good,—his courage under adversity and trial, his cheery philosophy,—his versatile genius are "shadowed" every month in *"Love's Medical Mirror."* It reflects nothing so much as his own mind,—his charming personality. Love is one of the brightest ornaments of the medical profession,—and the bright particular star in the firmament of the medical journal guild. Everybody knows him;—"not to know him were to argue one's self unknown." Fitz Green Halleck's immortal tribute to John Rodman Drake may be applied with truth and force to Love, changing only the tense of the verbs: "None know him but to love him; none name him but to praise." At the Association meetings he is always the center of an interested and admiring throng. He will make a ten-strike speech at the banquet, and then the fellows, who, like Oliver Twist, want more, will tote him on their shoulders to the nearest table, or in default of table,—chair, and make him speak again. Love has had a heap of trouble; he has been "misunderstood"—swindled; he has been persecuted, in fact, but he sheds trouble and worry like a duck does water, and practices that philosophy which he preaches so charmingly in the *Mirror.* In fact, he lives up to Davy Crockett's injunction: "Be sure you are right, and then go ahead." But like all philosophers, he is too "humane,"—he has a love for man which is not always appreciated, and sharpers take advantage of that weakness in his make-up which will never allow him, with all his courage, to say "no." We have often been tempted to copy some of his bright bits of "reflection,"—of philosophy; and we treat our readers now to a characteristic scrap or two.

(From *Love's Medical Mirror,* for June, 1900.

Nothing is so sustaining as a knowledge of duty done. Kipling wrote forcefully of the "Woman who could not Understand," but some one should write of the "Man who can not Understand" and who does not want to understand.

Toleration is one of the greatest words in the language.

Toleration adds so much to ones own happiness and its indulgence adds so much to the happiness of others, as to occasion regret that it is not universally cultivated.

After-all the hardest thing to tolerate is intolerance; all of which brings to mind the thought:

Shut the door on the day that is raining,
And let it go to, with a *slam,*
Bring wine, Mumm's Extra Dry, or Apollinaris,
  and open to no one,
But to the man who don't care a *d*——,
When he knows he's right.

Yes, the courage that inspires us to go on cheerfully, bravely, even merrily in the face of being misunderstood, to press on no matter what clouds may hover, thankful to God for the chance to live and love and labor for those we love and bring some sunshine into the lives of sorrowing souls, is the courage that makes life worth living.

What are we afraid of?

Something—every one of us—a great, misty, shadowy something that's always going to overwhelm us and almost never does.

Courage is the one virtue worth having. It is the one attribute which will carry a weak human being through this vale of tears creditably. Some persons don't believe in courage. They believe in cowardice.

I met a lady with a sensitive nature the other day. She has had trouble—most of us have. She has been bereaved—most of us have. She has lost her money—most of us have—and she is crushed. Simply crushed. So she wears mourning, with a veil like a shroud, and she grieves.

She lives all alone in a large house of her own and grieves. So useful. So edifying. There are people in trouble all around her. There are children to be fed, sick women to be nursed, old friends to be comforted, but she can't help it. She has no time to worry about things. She is too busy being crushed.

Her friends are very proud of her. They say she's such a delicate, sensitive creature.

This woman has a sister. The sister has suffered, too, suffered agonies of anguish that have left great furrowed scars across the sunlight of the world to her.

She has been deceived by the one she trusted, and there is no agony on earth like unto that. She has been sick and poor and deserted and forlorn.

But she has no time to grieve. She's too busy helping other people get well. She does not wear mourning. She takes great pains to dress as well as she can, so as to make a bright spot for some tired eyes to see. She makes it a point to be frivolous and light-hearted. She laughs a great deal.

People are much entertained by her. They ask her to visit them and when she's gone they say, "Isn't she a marvel; she's had trouble enough to kill any ordinary woman, but she doesn't feel things. Her poor sister, now, she's sensitive"—and they go and carry the poor sister some jelly and some flowers, and they pet her and pity her and she hugs her selfish, cowardly grief to her heart and is miserably proud of it.

I'm not proud of her. I'm ashamed of her, and I am proud of her sister, the woman who doesn't feel things, the woman who has put her own misery in the background and gone on and on and on.

---

**REFORM IN ASYLUM LAWS AND MANAGEMENT.**

Dr. B. M. Worsham, Superintendent of the Austin (State) Lunatic Asylum, recently made a trip through the Southern States, inspected the asylums and conferred with the superintendents as to the laws and the management of each institution. On his return he addressed an official letter to the governor of Texas, giving the results of his observations, and pointing out grave defects in our system which he urges should be at once remedied. His letter we reproduce elsewhere in this issue. It will be read with much interest, especially in connection with Dr. H. A. West's strong arraignment of the State government for bad laws and consequent inefficient administration of the State's great public charities, which paper was read at the recent meeting of the State Medical Association, and was published in our May number. Dr. Worsham appeals to the governor to recommend legislation to correct the evils he so clearly shows to exist, under our very primitive methods; the mode of commitment, especially, is barbarous. It remains to be seen whether his excellency will heed this appeal, or ignore it, as he is said to have done all petitions from the legislative committees of the State Medical Association. In light of the fact that the governor, though appealed to by the medical profession, has not recommended any legislation along these lines,—reform in our socalled "sanitary" and "medical laws," Dr. Worsham's allusion to the governor's "desire" and his "interest in having laws enacted" which would remedy these evils, struck us at first as a sly piece of sarcasm. But Worsham is not sarcastic; he is too deeply in earnest, as are all the good doctors in and out of the State Association. We were struck with the fact that Dr. Worsham left untouched the question of tenure of office by the medical officers of these institutions. That they are appointed for the most part for political reasons, and not because of known qualifications for the position, or because of experience in insane asylum management; and, as a rule, are turned out with each change of administration and new and raw men put in, is the greatest evil of our system. The advantage to the afflicted and to the State and to the public, of retaining in these offices, irrespective of political affiliation, men who have demonstrated their efficiency and given satisfaction, is too obvious for comment. But, as before remarked: How are you going to take the asylums out of politics? These appointments, as well as all others, are "patronage." They are a power; they mean the adhesion of the appointee to the interests of the "administration;" mean votes, and the average governor would sooner part with a front tooth than forego the privilege. But have we an "average governor" at this time? Some say he is above the average, and draw the inspiration of their hope from this belief.

## Figures and Tables

**Figure f1:**